# Involvement of the PI3K/Akt/NF-*κ*B Signaling Pathway in the Attenuation of Severe Acute Pancreatitis-Associated Acute Lung Injury by* Sedum sarmentosum* Bunge Extract

**DOI:** 10.1155/2017/9698410

**Published:** 2017-12-05

**Authors:** Yuepeng Jin, Lewei Liu, Bicheng Chen, Yongyu Bai, Fan Zhang, Qiang Li, Chongqing Lv, Hongwei Sun, Junjian Li, Sadman Rubby, Lihong Yang, Roland Andersson, Mengtao Zhou

**Affiliations:** ^1^Department of Surgery, The First Affiliated Hospital, Wenzhou Medical University, Wenzhou, Zhejiang Province, China; ^2^YueQing Affiliated Hospital of Wenzhou Medical University, YueQing People's Hospital, Yueqing, Zhejiang Province, China; ^3^Zhejiang Provincial Top Key Discipline in Surgery, Wenzhou Key Laboratory of Surgery, Department of Surgery, The First Affiliated Hospital, Wenzhou Medical University, Wenzhou, Zhejiang Province, China; ^4^Wenzhou Medical University, Wenzhou, Zhejiang Province, China; ^5^Shengli Oilfield Central Hospital, Dongying, Shandong Province, China; ^6^Department of Surgery, Clinical Sciences Lund, Lund University and Lund University Hospital, Sweden

## Abstract

*Sedum sarmentosum* Bunge possesses excellent anti-inflammatory properties and was used in the treatment of inflammatory diseases. The aim of the present study was to investigate the efficiency of* Sedum sarmentosum* Bunge extract (SSBE) on severe acute pancreatitis-associated (SAP-associated) acute lung injury (ALI) in rats and to explore the underlying mechanisms. Here, we used a sodium taurocholate-induced SAP rat model to determine the role of SSBE in ALI. During the course of pancreatitis, the expressions of phosphorylated phosphoinositide 3-kinases (PI3K)/protein kinase B (Akt) and nuclear factor-kappa B (NF-*κ*B) p65 in the lungs were upregulated. Meanwhile, a parallel increase in the levels of interleukin-1*β* (IL-1*β*), interleukin-6 (IL-6), and tumor necrosis factor-*α* (TNF-*α*) in the lungs was observed after the induction of SAP. Treatment with SSBE significantly reduced the expression of p-Akt and p-p65 in the lungs and attenuated the severity of SAP-associated ALI compared to the SAP group at 12 h and 24 h. In summary, this study showed that SSBE has beneficial effects on SAP-associated ALI, probably through the PI3-K/Akt signaling pathways by suppressing the NF-*κ*B activities.

## 1. Introduction

Severe acute pancreatitis is a potentially lethal inflammatory disease with variable involvement of the peripancreatic organs, and/or remote organ systems, by developing a systemic inflammatory response syndrome (SIRS) [1–7]. Among the systemic complications, ALI is the most frequent and potentially the most serious and may occur in 15% to 55% of SAP cases, including both ALI and the acute respiratory distress syndrome (ARDS) [8–10]. The associated mortality rate in SAP has been reported to be from 10% and up, where respiratory dysfunction (ALI and ARDS) is what mostly contributes to early mortality (i.e., during the first week) [5, 8, 11–14].

Although there are numerous factors involved in the development of SAP-associated ALI, inflammatory mediators have been reported to play a dominant role in the pathogenesis of SAP-associated ALI [7, 15]. Activation of inflammatory signaling pathways, such as PI3-K/Akt and NF-*κ*B, is involved as underlying molecular mechanisms in the development of ALI [7, 16–19]. The upstream regulator of NF-*κ*B involves PI3-K/Akt [16] signaling pathways. The underlying mechanisms of SAP-associated ALI are thus complex, which may explain the lack of specific pharmacological interventions, despite innumerous attempts over the years [14, 20–22].

*Sedum sarmentosum* Bunge is a perennial succulent herb frequently used in the treatment of inflammatory diseases in Oriental countries [23–25]. The complete genome of* Sedum sarmentosum *has been sequenced [26] and previous studies have revealed that* Sedum sarmentosum* Bunge possess anti-inflammatory, antiangiogenic, antinociceptive, and antifibrogenic properties [27–29]. Synthesis of monocyte and macrophage-mediated inflammatory factors can be decreased by* Sedum sarmentosum* Bunge [29]. Active chemical components in* Sedum sarmentosum* Bunge identified include megastigmane glycoside, quercetin, isorhamnetin, and kaempferide [27, 30].* Sedum sarmentosum* possess anti-inflammatory properties, and side effects seem to be limited [28]. In a previous preliminary study, we found anti-inflammatory effects and an attenuation of the morphological injury in the lungs in a rat model of SAP [31].

The purpose of the present study was to verify whether SSBE attenuated the severity of SAP-associated ALI. If this was the case, we aimed at elucidating the underlying mechanisms explaining the effect of SSBE in ALI.

## 2. Materials and Methods

### 2.1. Animals

The Laboratory Animal Center of Wenzhou Medical University (Wenzhou, China) supplied 84 healthy male Sprague-Dawley (SD) rats (weighing 250–300 g, 10–12 weeks old). The rats were maintained under specific pathogen-free (SPF) conditions and the temperature was kept at 20–22°C. A 12 h light-dark cycle was maintained, and all the rats were fed with a standard rat diet and water. All experiments were performed on animals after 12 h of fasting, but with free access to water.

### 2.2. Ethics Statement

The Institutional Animal Committee of Wenzhou Medical University, Wenzhou, China, approved the protocol for the animal experiment (Permit Number: wydw2013-0002). All animals received care in accordance with “Guide for the Care and Use of Laboratory Animals.” All surgical interventions were performed under chloral hydrate anesthesia.

### 2.3. Animals and Groups

Eighty-four male SD rats were randomly divided into 3 groups: control group (control, *n* = 28), SAP group (SAP, *n* = 28), and SSBE treatment group (SSBE, *n* = 28). Every group was divided into 4 subgroups, namely, 3, 6, 12, and 24 h subgroups (*n* = 7 animals/group).

### 2.4. Materials

The following materials had been purchased from the sources as mentioned below:* Sedum sarmentosum *Bunge extract (Xuancheng Baicao Plant Industry and Trade Co., Ltd., Anhui, China); sodium taurocholate (Sigma Chemical Company, St Louis, MO, USA); Rat ELISA Kit for detecting IL-1*β*, IL-6, and TNF-*α* (RapidBio Company, CA, USA); MPO Kit (JianCheng Biotechnology Research Institute, Nanjing, China); Akt and NF-*κ*B p65 and phosphorylated Akt and NF-*κ*B p65 antibodies (Cell Signaling Technology, Danvers, MA, USA); *β*-actin (Bioworld Technology, Louis Park, MN, USA).

### 2.5. The Animal Model for Induction of SAP

All procedures were performed in sterile conditions. The rats were anesthetized by subcutaneous injection of 10% chloral hydrate solution (0.3 mL/100 g). According to Aho et al. [32], SAP was induced by retrograde injection of 5% sodium taurocholate (1 ml/kg) into the main pancreatic duct through a microinjection pump at a speed of 0.1 ml/min. The control group had the same surgical procedure, but without the cannulation procedure and the retrograde injection of 5% sodium taurocholate.

### 2.6. Preparation and Administration of* Sedum sarmentosum* Bunge Extract

The extract protocol of* Sedum sarmentosum* Bunge has been shown by Bai et al. [29]. The whole plants were soaked in the tenfold distilled water for one hour and were used in the refluxing process of two hours. The extract was then filtrated and concentrated. Moreover, the drugs of the refluxing extract need further refluxing, filtration, and concentration. For animal experiments, 1 g of SSBE was dissolved in 10 ml of normal saline, resulting in a concentration of 100 mg/ml.

SSBE at a dose of 100 mg/kg was immediately administered subcutaneously and every 12 h following the establishment of the SAP model in the SSBE group. The 3, 6, and 12 h subgroups in the SSBE group were all administered with SSBE once. The 24 h subgroup in the SSBE group was administered with SSBE two times. Normal saline was administered in the same way as the other two groups.

### 2.7. Collection of Specimens

3, 6, 12, and 24 h after the induction of SAP, seven rats from each group were randomly selected to undergo abdominal incision under anesthesia. Blood and pancreatic and lung tissues were collected for further analysis.

### 2.8. Histopathological Analysis

Tissues from the pancreas and lungs were harvested and fixed in 10% formaldehyde solution, embedded in paraffin, sectioned, and stained with hematoxylin-eosin (H&E) for light microscopy. The pancreas pathological grading described by Schmidt et al. [33] was used, considering all factors, including edema, acinar necrosis, hemorrhage, fat necrosis, inflammation, and perivascular infiltration, when scoring the pancreatic injury in experimental SAP. Lung pathological scores were evaluated according to the method reported by Osman et al. [34]. An experienced pathologist examined the pathological sections unaware of the treatment groups, that is, in a blinded fashion.

### 2.9. Serum Amylase Activity and Levels of IL-1*β*, IL-6, and TNF-*α* in Lung Tissue

Serum amylase activity was determined by means of iodine-amylum colorimetry and expressed in U/L. Lung tissue was homogenized and centrifuged, and the supernatants expressed in pg/ml were collected to detect IL-1*β*, IL-6, and TNF-*α* by means of enzyme-linked immunosorbent assay (ELISA) using kits provided by the manufacturers.

### 2.10. Lung Wet/Dry Weight Ratio

The whole right lung of each rat was taken immediately after sacrifice, washed with PBS, and then weighed (wet weight). The tissues were dried for 72 h at 80°C in an electric oven and were then reweighed (dry weight). Tissue water content was determined by calculating the wet/dry weight ratio according to the formula: [water content = (wet weight − dry weight)/dry weight × 100%].

### 2.11. Lung MPO Activity

To carry out the assays, the supernatants of lung tissue were gathered after left lobectomy homogenization and centrifugation. Neutrophil sequestration within the lung was evaluated by quantitating tissue myeloperoxidase activity using a modification of the bromide-dependent chemiluminescence technique as described by Haqqani et al. [35].

### 2.12. Western Blot Analysis

For total cell extraction, tissue samples were homogenized in radio immunoprecipitation assay (RIPA) lysis buffer supplemented with protease inhibitor. The equal amounts of proteins were separated by 10% sodium dodecyl sulfate-polyacrylamide gel electrophoresis (SDS-PAGE) and transferred to a polyvinylidene difluoride (PVDF) membrane using transfer buffer at 350 mA for 70 minutes. Following the transfer of the protein, the membranes were blocked with 5% skim milk in tris-buffered saline with Tween-20 (TBST) for 2 h at room temperature and then incubated with one of the following primary antibodies overnight at 4°C: p-Akt (1 : 1000), Akt (1 : 1000), NF-*κ*B p65 (1 : 1000), or *β*-actin (1 : 5000). The membranes were then washed 3 times in TBST and incubated with a secondary antibody for 1 h at room temperature. Subsequently, they were washed 3 times for 10 min in TBST, and target bands were developed using enhanced chemiluminescence (ECL) and exposed on films. Densitometry was measured using the QuantityOne Version of 4.6.2 Image Software (Bio-Rad).

### 2.13. Immunohistochemistry

Contiguous 4 *μ*m sections were cut from paraffin embedded lung tissue for immunohistochemistry. Sections were blocked with goat serum and immunostained after deparaffinization and rehydration. Sections were then incubated overnight with a 1/200 dilution of primary antibody against p-Akt and p-p65 at 4°C, followed by incubation with secondary antibodies for 60 min at room temperature. The slides were developed with diaminobenzidine and counterstained with hematoxylin.

### 2.14. SSBE Side Effects Test

SSBE side effects were evaluated by checking serum alanine transaminase (ALT) and serum creatinine (Scr) levels in the liver and renal functions of male SD rats. Seven male SD rats were used as control (SSBE was not administered). Twenty-eight male SD rats were given SSBE subcutaneously at a dose of 100 mg/kg every 12 h. Blood samples were drawn from the control group as well (0 h group). Blood samples after SSBE administration were collected at 3 h, 6 h, 12 h, and 24 h after administration of SSBE. Serum ALT and serum Scr levels were determined using Beckman Coulter AU5821 automatic biochemistry analyzer (Beckman Coulter, Inc., Fullerton, CA, USA). The results were expressed in U/L and *μ*mol/L.

### 2.15. Statistical Analysis

Statistical Package for the Social Sciences (SPSS) software 18.0 (Chicago, IL, USA) was used to compute the mean ± standard deviation. Statistical analysis was analyzed by the one-way analysis of variance (ANOVA) followed by SNK-q test as a post hoc test. For all analyses, statistical significance was defined as *p* < 0.05.

## 3. Results

### 3.1. Morphological and Pathological Changes of Pancreas and Lungs

There were no morphological changes observed in the pancreas and lung tissue in the control group at any time points. Characteristic morphological features associated with acute pancreatitis, necrosis, calcic spots, hyperaemia, edema, color changes of the pancreas, and bloody ascitic fluid in the abdominal cavity were observed in the SAP group. In the SSBE group, calcic spots and bloody ascitic fluid in the abdominal cavity decreased significantly. Lung edema, local atelectasis, and pleural effusion were observed in the SAP group. These changes were relieved in the SSBE group at both 12 and 24 h.

**Figure 1:**
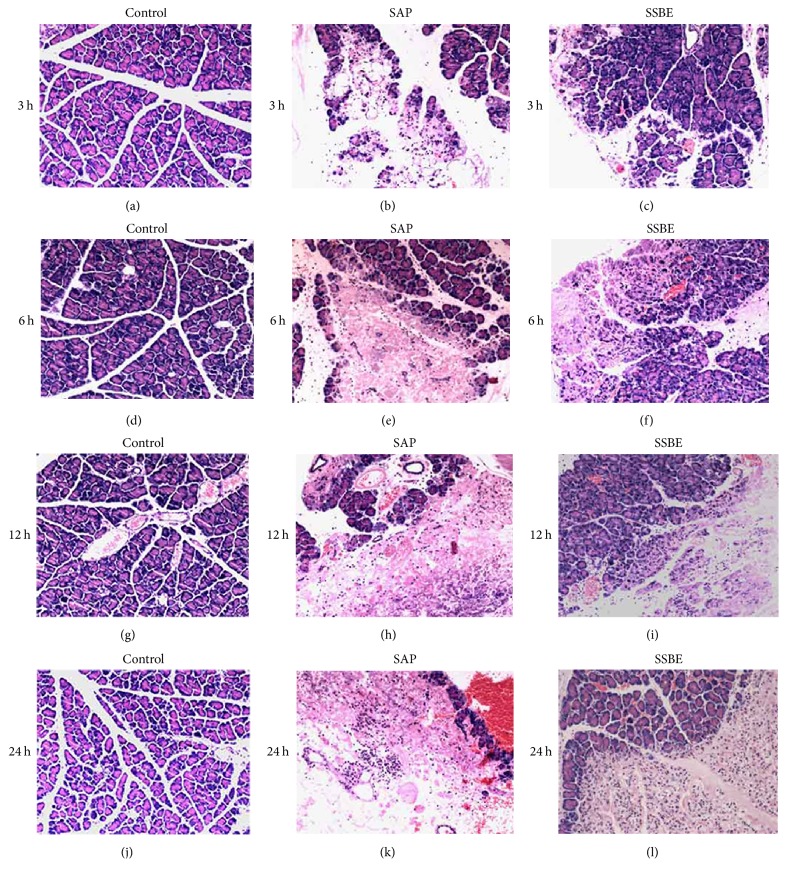
*Histopathological analysis of the pancreas in SAP-associated ALI rats*. Histopathological representation of the pancreas sections at different time points after sodium taurocholate induction of SAP. The representative haematoxylin-eosin stained sections of the pancreas at different time points from the control groups (a, d, g, and j), the SAP groups (b, e, h, and k), and the SSBE groups (c, f, i, and l). Original magnification: ×100.

**Figure 2:**
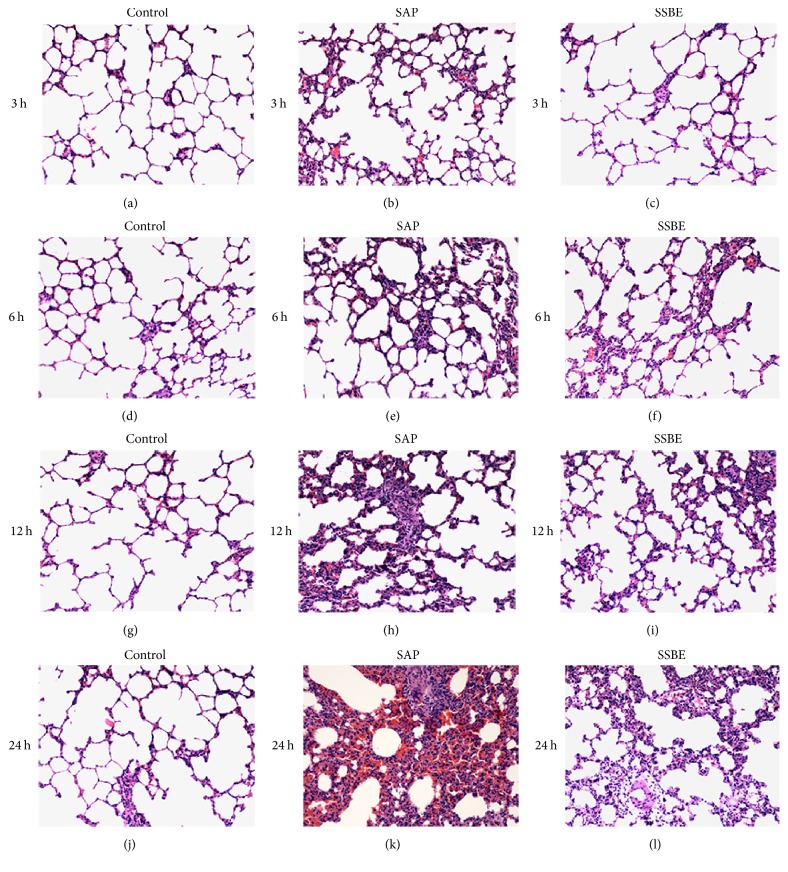
*Histopathological analysis of the lungs in SAP-associated ALI rats*. Histopathological representation of the lung sections at different time points after sodium taurocholate induction of SAP. The representative haematoxylin-eosin stained sections of the lung at different time points from the control groups (a, d, g, and j), the SAP groups (b, e, h, and k), and the SSBE groups (c, f, i, and l). Original magnification: ×100.

Pathological examination was normal in the control group. Under the microscope, acinar cell necrosis, cytoplasmic vacuolization, edema, inflammatory cell infiltration, and hemorrhage were observed in the pancreas from 3 h after induction, with increasing severity after 6, 12, and 24 h. Alveolar wall thickening and infiltration of inflammatory cells in the lungs were shown from 3 h after pancreatitis induction and were aggravated at 6, 12, and 24 h. Mild hemorrhage was noted in the lungs from 6 h after the induction of pancreatitis and was aggravated with time. These changes in the pancreas and lungs were alleviated in the SSBE group compared to the SAP group at both 12 and 24 h (Figures 1 and 2).

**Figure 3:**
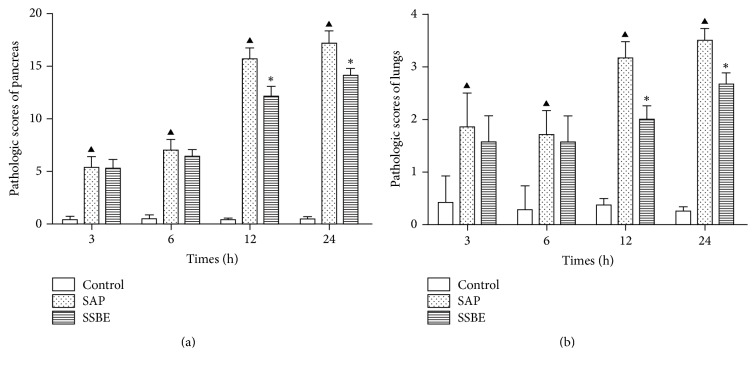
*SSBE improved the pathological scores of the pancreas and lungs*. The scores of the pancreas and lungs in the SSBE group were significantly lower compared to the SAP group at 12 and 24 h. (^▲^*p* < 0.05 SAP group versus control group; ^*∗*^*p* < 0.05 SSBE group versus SAP group.)

The pathological scores of the pancreas and lung tissue in the SSBE group were lower compared to the SAP group at 12 and 24 h (*p* < 0.05; Figure 3).

### 3.2. Serum Amylase Activity and Lung Wet/Dry Weight Ratio

**Figure 4:**
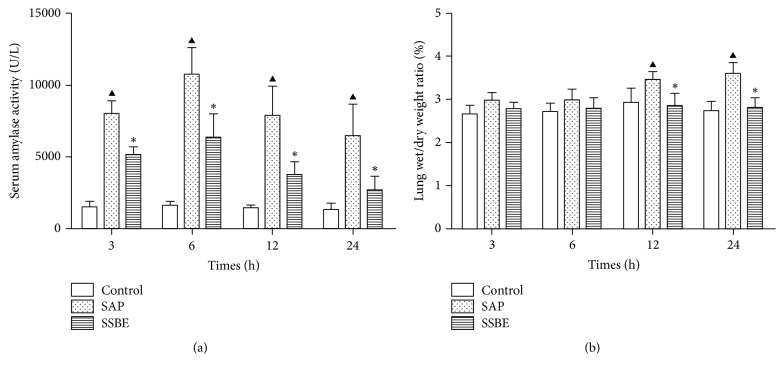
*Serum amylase activity and lung wet/dry weight ratio*. The SSBE group had lower levels of serum amylase at all four time points as compared to the SAP group. The lung wet/dry weight ratio in the SSBE group was lower as compared to the SAP group at 12 and 24 h. (^▲^*p* < 0.05 SAP group versus control group; ^*∗*^*p* < 0.05 SSBE group versus SAP group.)

Serum amylase activity increased in the SAP group and the SSBE group following the induction of SAP. Serum amylase activity in the SSBE group was significantly lower than in the SAP group at 3, 6, 12, and 24 h (*p* < 0.05; Figure 4).

Lung wet/dry weight ratio was manifested over time. It was reduced in the SSBE group compared to the SAP group at 12 and 24 h (*p* < 0.05). A 3.62% of lung wet/dry weight ratio in the SAP group was shown after 24 h compared to 2.81% in the SSBE group (*p* < 0.05; Figure 4).

### 3.3. Lung MPO Activity and Levels of IL-1*β*, IL-6, and TNF-*α* in Lung Tissue

**Figure 5:**
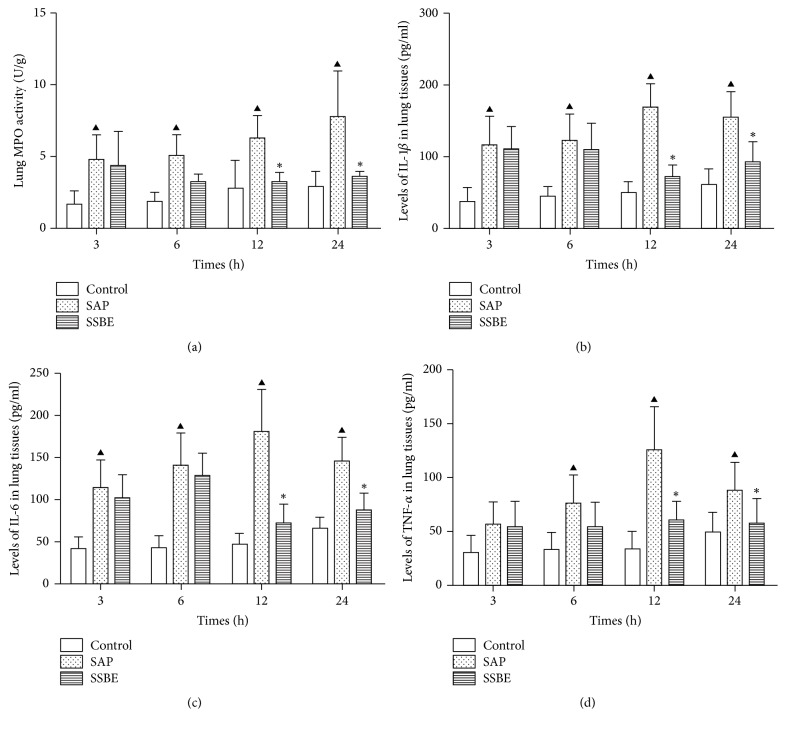
*Lung MPO activity and levels of IL-1β, IL-6, and TNF-α in the lung tissue*. The SSBE group had a lower lung MPO activity and levels of IL-1*β*, IL-6, and TNF-*α* in the lung tissue at 12 and 24 h time points as compared to the SAP group. No significant changes of lung MPO activity, IL-1*β*, IL-6, and TNF-*α* in lung tissue were found in the control group. (^▲^*p* < 0.05 SAP group versus control group; ^*∗*^*p* < 0.05 SSBE group versus SAP group.)

Neutrophil sequestration in the lungs was quantified by measuring the lung MPO activity. As shown in Figure 5, sodium taurocholate-induced pancreatitis-associated ALI was associated with an increase in lung MPO activity at 3, 6, 12, and 24 h. Increased lung MPO activity indicated that neutrophils were sequestered within the lungs. Treatment with SSBE decreased the lung MPO activity after the induction of pancreatitis at 12 and 24 h (*p* < 0.05; Figure 5).

To determine the expression of proinflammatory cytokines in the lungs, IL-1*β*, IL-6, and TNF-*α* were evaluated. An increased expression of the proinflammatory cytokines IL-1*β*, IL-6, and TNF-*α* in the lung tissue in the SAP group was seen as compared to the control group; though the expression of TNF-*α* did not significantly differ between the two groups at 3 h. The peak expression of L-1*β*, IL-6, and TNF-*α* in the lung tissue occurred at 12 h after the induction of pancreatitis. In contrast, treatment with SSBE significantly reduced the production of L-1*β*, IL-6, and TNF-*α* at 12 and 24 h (*p* < 0.05; Figure 5). However, the levels of IL-1*β*, IL-6, and TNF-*α* in the lung tissue did not significantly differ between the SAP group and the SSBE group at 3 and 6 h (*p* > 0.05). These data indicate that SSBE decreased the expression of proinflammatory cytokines in the lungs and the onset time of SSBE was probably after 12 h.

### 3.4. Expression of p-Akt/Akt Protein in the Lung Tissue

**Figure 6:**
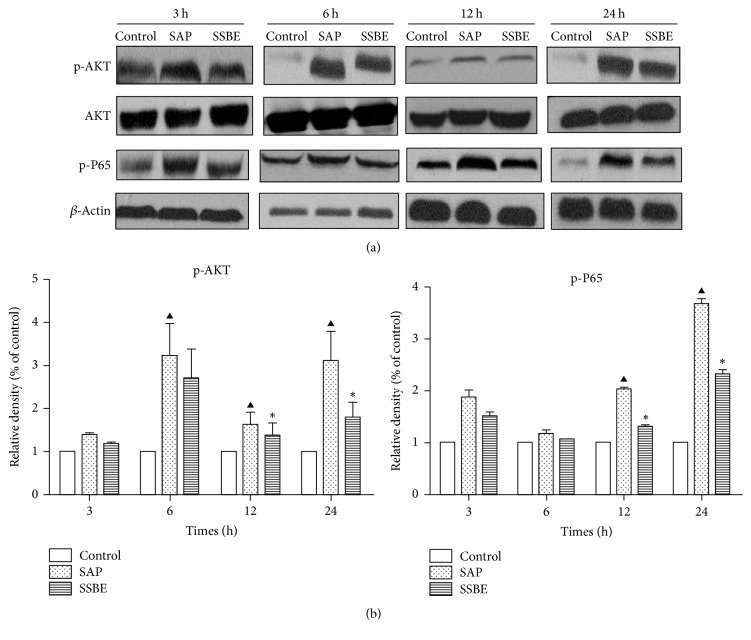
*Western blot analysis of p-Akt/Akt and p-p65 protein levels in lungs at time points indicated*.* (a) Western blot results and (b) densitometric measurements*. (a) Western blot analysis of the protein expression of p-Akt and p-p65 in lung tissue at different time points in the control group, the SAP group, and the SSBE group. (b) The graph shows densitometric measurements of Western blot. Activation of Akt was shown as the relative ratio between phosphorylated and total kinase. Activation of p65 was shown as the relative ratio of p-p65 to *β*-actin. (^▲^*p* < 0.05 SAP group versus control group; ^*∗*^*p* < 0.05 SSBE group versus SAP group.)

**Figure 7:**
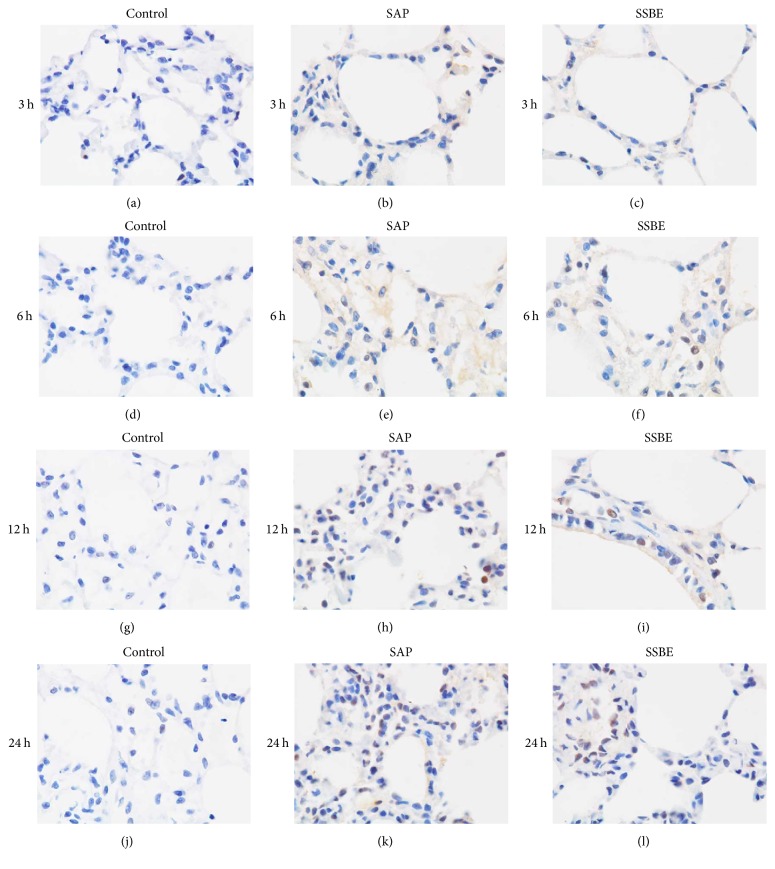
*Immunohistochemical staining for p-Akt proteins expression in the lungs*. Positive cells were stained dark brown. The control groups (a, d, g, and j): negative expression in the control groups at different time points. The SAP groups (b, e, h, and k): weak positive expression at 3 h and 6 h and strong positive expression in the SAP groups at 12 and 24 h. The SSBE groups (c, f, i, and l): positive expression at 12 and 24 h and weaker positive expression in the SSBE group compared to the SAP group at 12 and 24 h. Weak positive expression at 3 and 6 h, which was similar to the SAP groups at the same time points. Original magnification: ×1000.

The protein expression of p-Akt/Akt in the lung tissue was detected by Western blot analysis in our study. The protein expression of p-Akt in the SAP group increased significantly at the 6, 12, and 24 h time points compared to the control group. Treatment with SSBE decreased the protein expression of p-Akt in the lung tissue at 12 and 24 h (*p* < 0.05) but did not decrease at 3 and 6 h (*p* > 0.05) (Figure 6). Furthermore, immunohistochemistry of p-Akt expression yielded similar results to Western bolt analysis (Figure 7).

### 3.5. Expression of NF-*κ*B p-p65 in the Lung Tissue

**Figure 8:**
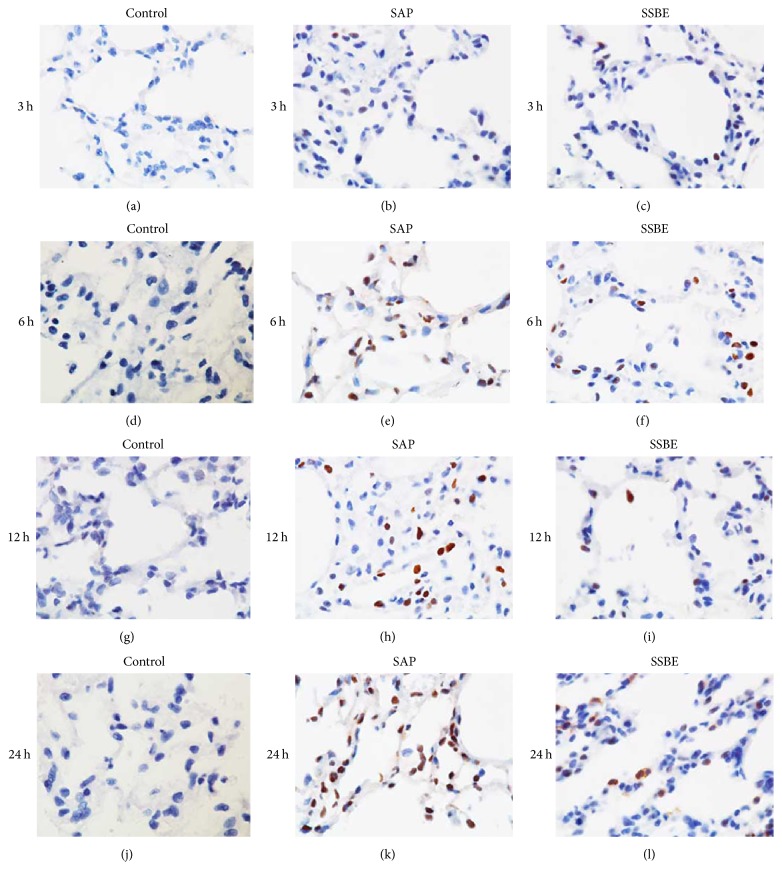
*Immunohistochemical staining for p-p65 proteins expression in the lungs*. Positive cells were stained dark brown. The control groups (a, d, g, and j): negative expression in the control groups at different time points. The SAP groups (b, e, h, and k): weak positive expression at 3 h and 6 h and strong positive expression in the SAP groups at 12 and 24 h. The SSBE groups (c, f, i, and l): positive expression at 12 and 24 h and weaker positive expression in the SSBE group compared to the SAP group at 12 and 24 h. Weak positive expression at 3 and 6 h, which was similar to the SAP groups at the same time points. Original magnification: ×1000.

Immunohistochemistry was performed to detect the expression of NF-*κ*B p-p65 in the lung tissue in each group. A negative expression of NF-*κ*B p-p65 was observed in the lung tissue in the control group at all time points. A weak positive expression in the SAP group was seen at 3 and 6 h, with increasing positive expression after 12 and 24 h. Treatment with SSBE decreased the expression of NF-*κ*B p-p65 in the lung tissue at 12 and 24 h. The expression of NF-*κ*B p-p65 in the lung tissue was similar between the SAP and SSBE groups at 3 and 6 h (Figure 8).

Furthermore, Western blot analysis was used to investigate the expression of NF-*κ*B p-p65 in the lung tissue. As shown in Figure 6, the expression of NF-*κ*B p-p65 significantly increased in the SAP group at 12 and 24 h compared to the control group, while treatment with SSBE resulted in decreased protein expression of p-p65 at 12 and 24 h (*p* < 0.05).

### 3.6. The Mortality Rate and Side Effects

The mortality rate in this research was zero. At a dose of 100 mg/kg q12 h there were no side effects observed as shown in Table 1.

## 4. Discussion

Previous pharmacological studies have shown that SSBE possesses excellent anti-inflammatory properties [27–29]. However, the effects of SSBE in experimental SAP-associated ALI have not been elucidated. Understanding the mechanisms underlying SSBE anti-inflammatory properties would help to improve both understanding and the potential development of therapies directed towards SAP-associated ALI. PI3K/Akt and NF-*κ*B are downstream inflammatory signaling pathways, which seem involved in the development of ALI [7, 16–19]. In the present study, we found that SSBE downregulated the activation of Akt and NF-*κ*B p65 in the lungs, contributing to the decrease of proinflammatory cytokines in sodium taurocholate-induced ALI rats model at later stages (12 and 24 h).

Acute lung injury is the most pertinent manifestation of extra-abdominal organ dysfunction in SAP, not at least an important cause of especially early (the first week) mortality during the course of SAP [36]. It is considered that high concentrations of inflammatory mediators and activation of inflammatory cells play an important role in the initiation and progression of ALI [7, 15, 37]. The inflammatory mediators augment the inflammatory response, which stimulate the inflammatory cascade, leading to the systemic complications of acute pancreatitis. As a result, they cause the systemic inflammatory response syndrome (SIRS), acute respiratory distress syndrome (ARDS), and the multiple organ dysfunction syndrome (MODS). However, effective therapies for preventing development of ALI in pancreatitis are still lacking. Therefore, there is an urgent need for a potential beneficial pharmacological intervention.

In this study, SAP-associated ALI was induced by sodium taurocholate. Lung injury was proven, showing pulmonary inflammatory cell infiltration and alveolar wall thickening, as has been previously also demonstrated [31]. Sequestration of inflammatory cells, particularly neutrophils, within the lungs contributes to the development and severity of pancreatitis-associated lung injury [38]. MPO accounts for 3–5% of the total neutrophil proteins, making it the dominant neutrophil oxidant-generating system. Our results showed that the lung MPO activity was markedly upregulated during SAP-associated ALI. This is the most likely explanation for the neutrophil sequestration in the lungs. The neutrophils present in the lungs during ALI produce cytokines, resulting in an increased expression of proinflammatory cytokines, including IL-1*β*, IL-6, and TNF-*α* in the lungs. The neutrophil infiltration and the increased levels of proinflammatory cytokines in the lungs aggravated the ALI. The currently available data concerning the model of SAP-associated ALI, including the increased pulmonary inflammatory cell infiltration, and alveolar wall thickening, coupled with the elevation of proinflammatory cytokines, was indicative of the development of ALI after sodium taurocholate induction [39–41]. Treatment with SSBE as demonstrated in the present study attenuated the pathological pulmonary injury scores, the wet/dry weight ratio, MPO activity, and proinflammatory cytokines in the lungs, indicating that SSBE reduced the severity of sodium taurocholate-induced lung injury. However, it was noticed that the effect of SSBE in SAP-associated ALI was gradually increasing, with a peak at about 12 h after induction.

Several reports have demonstrated that PI3-K/Akt and NF-*κ*B signaling pathways participate in the regulation of inflammatory mediators, thus leading to acute lung injury [16, 42–45]. In order to improve insights in the underlying mechanisms of SSBE in experimental ALI, we measured the levels of phosphorylated Akt and NF-*κ*B p65 in the lungs. Previous studies have shown that PI3-K, via the activation of Akt, can lead to an increase of the nuclear translocation and transcriptional activity of NF-*κ*B [16, 46–48], which has a critical role in regulating the expression of cytokines, as well as the proinflammatory mediators involved in ALI. Its activation has a key influence on the pathogenesis of SAP [49], and, hence, inhibition of NF-*κ*B can improve the survival of rats with pancreatitis [50]. Activation of Akt and NF-*κ*B p65 in the lungs was measured by Western blot analysis and immunohistochemistry methods after induction of pancreatitis. Meanwhile, a parallel increase in the levels of proinflammatory cytokines (IL-1*β*, IL-6, and TNF-*α*) in the lungs was observed after the induction of SAP. These finding may indicate that Akt and NF-*κ*B signaling pathways probably have been activated during the early stage of pancreatitis-associated lung injury, leading to the release of proinflammatory cytokines. Our results showed that treatment with SSBE can downregulate the expression of phosphorylated Akt and NF-*κ*B p65 in the lungs at later stages (12 and 24 h), contributing to the decrease of proinflammatory cytokines. Combined with previous reports, supporting our findings, it is implied that the underlying mechanisms of SSBE in SAP-associated ALI may, at least partly, be mediated via Akt and NF-*κ*B signaling pathways.

Due to the complexity of the chemical components of SSBE, the isolated component(s) responsible for contributing to the anti-inflammatory activity still need to be further studied and elucidated. The dosage and concentration of SSBE for the treatment of acute pancreatitis-associated ALI have not been reported previously. In previous studies,* Sedum sarmentosum* methanol in higher doses was associated with the best anti-inflammatory properties [28]. In our preliminary studies (data not shown), we had used a lower dosage of SSBE (25 mg/kg, q12 h), but the activity of serum amylase did not decrease and no change was noted in the pathological scores, so a higher dosage of SSBE (100 mg/kg, q12 h) was chosen in the present study.

## 5. Conclusion

In summary, we demonstrated that SSBE shows potent anti-inflammatory properties, and it is potentially capable of improving SAP-associated ALI, probably through the PI3-K/Akt signaling pathways by suppressing the NF-*κ*B activities. As a result, the production of proinflammatory cytokines in the lungs decreased, an effect beneficial in acute lung injury. These findings provide new insights into the role played by SSBE in acute pancreatitis-associated ALI, suggesting its potential value once the exact and most effective compounds have been identified.

## Figures and Tables

**Table 1:** Serum alanine transaminase (ALT) and serum creatinine (Scr) levels after administering SSBE at different time points.

	0 h (control)	3 h	6 h	12 h	24 h
ALT (U/L)	57.00 ± 2.51	57.14 ± 1.95	59.14 ± 9.10	54.14 ± 6.54	55.71 ± 7.32
Scr (*μ*mol/L)	37.29 ± 3.99	35.43 ± 4.31	37.86 ± 3.13	36.14 ± 6.04	37.14 ± 5.46

Seven male SD rats were used as control (SSBE was not administered). Twenty-eight male SD rats were given SSBE subcutaneously at a dose of 100 mg/kg every 12 h. Blood samples were also drawn from the control group (0 h group). Blood samples after SSBE administration were collected at 3 h, 6 h, 12 h, and 24 h after administration of SSBE.
